# Evermore

**Published:** 1863-10

**Authors:** 


### EVERMORE.

I BEHELD a golden portal in the visions of my slumber,
And through it streamed the radiance of a never-setting day;
While angels tall and beautiful, and countless without number,
Were giving gladsome greeting to all who came that way.
And the gates forever swinging, made no grating, no harsh ringing,
Melodious as the singing of one that we adore;
And I heard a chorus swelling, grand beyond a mortal's telling,
And the burden of that chorus was Hope's glad word—Evermore?

### EVERMORE.

As I gazed and listened, came a slave all worn and weary,
His fetter-links blood-crusted, his dark brow clammy damp,
His sunken eyes gleamed wildly, telling tales of horror dreary,
Of toilsome strugglings through the night amid the fever swamp.
Ere the eye had time for winking, ere the mind had time for thinking,
A bright angel raised the sinking wretch, and off his fetters tore;
Then I heard the chorus swelling, grand beyond a mortal's telling:
"Pass, brother, through our portal, thou'rt a freeman evermore!"

And as I gazed and listened, came a mother wildly weeping—
"I have lost my hopes forever, one by one they went away;
My children and their father the cold grave hath in its keeping,
Life is one long lamentation, I know nor night nor day!"
Then the angel softly speaking: "Stay, sister, stay thy shrieking,
Thou shalt find those thou art seeking beyond that golden door!"
Then I heard the chorus swelling, grand beyond a mortal's telling:
"Thy children and their father shall be with thee evermore!"

And as I gazed and listened, came one whom desolation
Had driven like a helmless bark from infancy's bright land;
Who ne'er had met a kindly look—poor outcast of creation—
Who never heard a kindly word, nor grasped a kindly hand.
"Enter in, no longer fear thee; myriad friends are there to cheer thee;
Friends always to be near thee, there no sorrow sad and sore!"
Then I heard the chorus swelling, grand beyond a mortal's telling:
"Enter, brother, thine are friendship, love and gladness evermore!"

As I gazed and listened, come a cold, blue-footed maiden,
With cheeks of ashen whiteness, eyes filled with lurid light;
Her body bent with sickness, her lone heart heavy laden;
Her home had been the roofless street, her day had been the night.
First wept the angel sadly, then smiled the angel gladly,
And caught the maiden madly rushing from the golden door;
Then I heard the chorus swelling, grand beyond a mortal's telling:
"Enter, sister, thou art pure, and thou art sinless evermore!"

I saw the toiler enter to rest for aye from labor;
The weary-hearted exile there found his native land;
The beggar there could greet the king as an equal and a neighbor;
The crown had left that kingly brow, the staff the beggar's hand.
And the gate forever swinging, made no grating, no harsh ringing,
Melodious as the singing of one that we adore;
And the chorus still was swelling, grand beyond a mortal's telling,
While the vision faded from me with the glad word—"Evermore!"

—*Edinburgh Guardian.*

